# Transient cortical blindness after diagnostic cerebral angiography – A single center report

**DOI:** 10.1016/j.heliyon.2024.e29256

**Published:** 2024-04-04

**Authors:** A. Iancu, O. Nikoubashman, S. Tafelmeier, E. Kesseler, M. Wiesmann

**Affiliations:** Department of Neuroradiology, University Hospital RWTH Aachen, Pauwelsstr. 30, 52074, Aachen, Germany

## Abstract

**Background:**

Transient cortical blindness (TCB) has been reported as a complication after diagnostic cerebral angiography in 0.3–1% of cases. Our aim was to observe the frequency of TCB after diagnostic cerebral angiography over a period of 11 years using only hypo-osmolar, nonionic contrast agents and following a protocol to reduce both the total volume of injected contrast agent and the number of angiography series obtained.

**Methods:**

We retrospectively included all 2431 patients who received diagnostic cerebral angiographies at our institution. Primary outcome measure was the occurrence of TCB after diagnostic cerebral angiography, hypothesizing that the occurrence of TBC depends on the volume of contrast agent and angiography of the vertebrobasilary arteries.

**Results:**

Over the analyzed time period of 11 years, we did not observe a single case of TCB following diagnostic cerebral angiography. The median contrast volume used was 100 ml (IQR, 100–200), ranging from 15 ml to 500 ml. In our cohort, 61.5% of patients received a selective catheterization of the vertebrobasilary territory. In 99.8% of angiographies iopamidol was used a contrast agent.

**Conclusion:**

Our results indicate that following to certain aspects of the angiography protocol (using the hypoosmolar, non-ionic contrast agent iopamidol and reducing the number of catheterized vessels and angiography series to a diagnostic minimum) the frequency of transient cortical blindness as a complication of diagnostic cerebral angiography considerably can be very low.

## 1 Introduction

Neuroangiography is a common diagnostic tool in the workup of neurovascular diseases. Because it is invasive, it is associated with a variety of complications. One of these complications is transient cortical blindness (TCB), firstly reported in 1970 after coronary angiography [[1], [2], [3]]. This clinically dramatic but reversible complication has been reported to occur in up to 1% of patients undergoing cerebral angiography and 0.06% of patients undergoing cardiac catheterization [[4], [5], [6]]. TCB is defined as an impaired or complete loss of vision with normal fundoscopic examination, unaltered papillary reflexes and intact function of the extraocular muscles. The onset of the symptoms occurs usually within the first 12 h after angiography and can be accompanied by other neurological symptoms. Symptoms usually disappear within the first 12 h but can last up to 5 days [7]. It has been hypothesized that TCB is a manifestation of posterior reversible encephalopathy syndrome (PRES) and triggered by large amounts of contrast agents, especially when ionic contrast agents with high osmolarity are being used [8], and when angiography is performed in the posterior circulation [9,10].

Our aim was to retrospectively assess the frequency of TCB following diagnostic cerebral angiography at our institution over a period of 11 years.

## 2 Material and methods

We retrospectively included all patients who received diagnostic cerebral angiographies in the Department of Diagnostic and Interventional Neuroradiology of the RWTH University Hospital Aachen between January 2010 and December 2020. The patients that have undergone an intervention during the same session were excluded from the study. This analysis was approved the Ethics Committee of our institution. Need for individual patient consent was waived by our Ethics Committee.

### 2.1 Digital subtraction angiography

Our standard procedure was as follows: Angiographies are usually performed on a biplane angiography system (Siemens Artis zee, Siemens Healthineers, Forchheim, Germany). Diagnostic angiographies were always performed by trained neuroradiologists. 4F or 5F catheters of different shapes were used via a femoral and sometimes radial access site depending on the patient's vascular anatomy. Only vessels relevant for the respective diagnosis were probed and injected. All angiography material was flushed with heparinized saline solution (1000 IU heparin diluted in 1000 mL saline). The used contrast agent in 2426 of 2431 patients (99.8%) was iopamidol (Solutrast 300, Bracco Imaging, Milano, Italy), which is a hypoosmolar non-ionic contast agent with an iodine concentration of 300 mg/l, an osmolality of 616 mosm/kg H_2_0, an osmolarity of 436 mosm/l, and a pH-value between 6.5 and 7.5. Only five patients received iopromide (Ultravist 300, Bayer Healthcare Pharmaceuticals Inc., Wayne, NJ). Injected contrast agents were diluted with saline solution (8:2). In our Department injections for rotational angiograms are routinely performed as hand injections. Therefore, all injections in this cohort were hand injections. One day prior to angiography and on the day after cerebral angiography, all patients at our institution receive a thorough neurological examination. It is routine clinical practice at our institution to evaluate all patients prior to angiography and perform additional hydration 12 h before angiography if indicated (i.e. in cases of reduced glomerular filtration rate or polycythemia).

## 3 Methods

Primary outcome measure was the occurrence of TCB after diagnostic cerebral angiography, hypothesizing that the occurrence of TCB depends on the volume of contrast agent and angiography of the vertebrobasilary arteries. The amount of injected contrast agent was retrospectively derived from the documented number of used bottles of contrast agent; hence indicated volumes are maximum volumes, and injected volumes were lower than reported. The reported contrast volume is before dilution with saline.

Parametric values are indicated as mean ± standard deviation (SD) and non-parametric values are indicated as median and interquartile range (IQR).

All clinical examinations after the cerebral angiography were performed by an experienced neurological doctor. The patients were assessed on whether they experienced any kind of partial or complete visual impairment.

## 4 Results

We analyzed 2431 consecutive patients who received diagnostic cerebral angiography. 54.6% (1327) were female and mean age of patients was 58 ± 17.3 years, ranging from 2 to 90 years.

Median contrast volume was 100 ml (IQR, 100–200), ranging from 15 ml to 500 ml. A total of 61.5% of patients (1440/2431) received selective catheterization of the vertebrobasilar territory with injection of the contrast agent directly into the vertebral arteries.

Over the analyzed time period of 11 years, not a single patient developed TCB following diagnostic cerebral angiography.

## 5 Discussion

The most surprising aspect of our analysis is that there were no cases of transient cortical blindness (TCB) in a cohort of 2431 consecutive patients who underwent cerebral angiography over a period of 11 years. The largest multicenter study regarding this possible complication was published in 2019 by Li M et al. with a reported complication rate of 0.35% in a total cohort of 5126 patients. They also reported using a nonionic low-osmolar contrast material [11]. Having taken this study into consideration and given the reported prevalence of 0.3–1% in the literature, we would have expected 7–24 cases [7,9,11].

The pathophysiology of TCB is unclear with many theories being proposed. One of these theories is a direct neurotoxic effect on the blood-brain barrier of the occipital lobe [5,12]. As previously described by Yazici et al. [2] and Chen et al. [3], the injection of hyperosmolar solutions can disrupt the blood-brain barrier. Following disruption of the blood-brain barrier interstitial edema develops which might then impair neuronal function until the blood-brain barrier is restored and the amount of interstitial fluid is restored to normal. Saigal et al. reported that there appears to be a similarity between transient cortical blindness and posterior reversible leukoencephalopathy after injection of contrast agent. The concept of related entities emerges from comparable MR imaging aspects between the two, suggesting that they have a common pathophysiology injuring the neurons within the occipital cortex [9].

Data in the literature suggest that both hypertonicity of the used contrast agent and high amounts of injected volume may promote the occurrence of TCB [5,7,8]. The fact that TCB is less frequent in cardiac angiography, which comes without direct injection of contrast agent into the cerebral arteries, suggests that high local concentrations are needed to induce TCB. However, there have been various case reports of transient cortical blindness that occurred after injection ranging from as little as 25 mL up to 400 mL of contrast agent, indicating that volume alone is not the only risk factor [2,3,12,13].

It is believed that TCB is rather promoted by ionic than non-ionic contrast agents: non-ionic agents are supposed to be safer than ionic contrast agents because of the lesser toxic impact they have on the endothelial cells of blood vessels. The reported incidence of 0.3–1% with the use of non-ionic contrast agents such as ioxaglate, iopamidol or iohexol increased up to 4% when ionic contrast agents with high osmolarity were being used [8]. Supporting this, there are only few reported cases of TCB in coronary angiography with non-ionic contrast agents such as iohexol or iodixanol [3,13].

Several factors may have contributed to the unexpected finding that we did not observe a single case of TCB in 2431 consecutive cerebral angiographies [1]. Only non-ionic contrast agents were used [2]. Contrast agents were usually diluted with saline solution (8:2) before injection thereby further reducing osmolarity [3]. The total volume of contrast agent required was considerably reduced because almost all angiographies were performed using a biplane angiography unit [4]. At our institution we have implemented the practice that angiography series are obtained only from those vessels, which are definitely required for diagnosis, instead of performing "standard" 4-vessel angiographies. This results in a reduction of both the total volume of required contrast, and the number of cases in which vessels of the posterior circulation need to be injected.

Nevertheless, the fact that Li et al. [11] observed TCB in 18 cases (0.35%) of 5126 patients whereas we did not observe a single case in 2431 patients is remarkable. If tested using a chi-squared test this difference would be significant (p < 0.01). Li et al. found in their analysis that logistic regression analysis identified only contrast agent dose and contrast agent injected into posterior circulation as independent predictive factors for transient cortical blindness. However, both the mean contrast agent dose they report (99.9 ml) and their rate of vertebral injections (59.4%) appear to be comparable to our results. Both in the study of Li et al. and our cohort only nonionic low-osmolar contrast agents were used. Li et al. used ioversol (Optiray) as contrast agent, whereas in our study iopamidol (Solutrast 300) was used almost in all cases. It seems premature, however, to conclude that the reduced frequency of TCB we observed was caused by the type of contrast agent. This needed to be verified in a randomized study.

Our results indicate that following to certain aspects of the angiography protocol (using the hypoosmolar, non-ionic contrast agent iopamidol and reducing the number of catheterized vessels and angiography series to a diagnostic minimum) the frequency of transient cortical blindness as a complication of diagnostic cerebral angiography considerably can be very low.

## Limitations

A major limitation of our analysis is its retrospective approach, which carries the risk for a selection and detection bias. Another limitation is the single-centre design. Although our study includes 2431 cases, sample size limitations must always be considered when rare complications are analyzed.

## Declarations

The study was conducted in accordance with the Declaration of Helsinki, and approved by the Ethics Committee of the University Hospital RWTH Aachen (EK055/21, February 11, 2021). Patient consent was waived by our Ethics Committee due to the retrospective nature of our analysis.

The Authors declare that there is no conflict of interest.

## Data availability statement

Data will be made available on request.

## CRediT authorship contribution statement

**A. Iancu:** Writing – original draft, Methodology, Investigation, Formal analysis, Data curation, Conceptualization. **O. Nikoubashman:** Writing – review & editing, Supervision, Methodology. **S. Tafelmeier:** Data curation, Formal analysis, Investigation, Writing – review & editing. **E. Kesseler:** Writing – review & editing, Investigation, Data curation, Formal analysis. **M. Wiesmann:** Writing – review & editing, Validation, Supervision, Resources, Project administration, Methodology, Conceptualization.

## Declaration of competing interest

The authors declare that they have no known competing financial interests or personal relationships that could have appeared to influence the work reported in this paper.
